# Hemodynamic and Non-Hemodynamic Components of Cardiac Remodeling in Primary Aldosteronism

**DOI:** 10.3389/fendo.2021.646097

**Published:** 2021-04-19

**Authors:** Chien-Ting Pan, Xue-Ming Wu, Cheng-Hsuan Tsai, Yi-Yao Chang, Zheng-Wei Chen, Chin-Chen Chang, Bo-Ching Lee, Che-Wei Liao, Ya-Li Chen, Lung-Chun Lin, Yi-Ru Chang, Chi-Sheng Hung, Yen-Hung Lin

**Affiliations:** ^1^ Department of Internal Medicine, National Taiwan University Hospital Yun-Lin Branch, Yun-Lin, Taiwan; ^2^ Department of Internal Medicine, National Taiwan University Hospital and National Taiwan University College of Medicine, Taipei, Taiwan; ^3^ Cardiovascular Center, National Taiwan University Hospital, Taipei, Taiwan; ^4^ Department of Internal Medicine, Taoyuan General Hospital, Taoyuan, Taiwan; ^5^ Department of Internal Medicine, National Taiwan University Hospital, JinShan Branch, New Taipei, Taiwan; ^6^ Cardiology Division of Cardiovascular Medical Center, Far Eastern Memorial Hospital, New Taipei City, Taiwan; ^7^ Center of General Education, Chihlee University of Technology, New Taipei City, Taiwan; ^8^ Department of Medical Imaging, National Taiwan University Hospital and National Taiwan University College of Medicine, Taipei, Taiwan; ^9^ Department of Internal Medicine, National Taiwan University Hospital Hsin-Chu Branch, Hsin-Chu, Taiwan

**Keywords:** primary aldosteronism, aldosterone producing adenomas, aldosterone (ALDO), cardiac remodeling, left ventricular hypertrophy (LVH), inappropriately excessive left ventricular mass

## Abstract

**Objectives:**

Patients with primary aldosteronism (PA) have cardiac remodeling due to hemodynamic and non-hemodynamic causes. However, component analysis of cardiac remodeling and reversal in PA patients is lacking. We investigated components of cardiac remodeling and reversal after adrenalectomy in patients with aldosterone-producing adenoma (APA).

**Methods:**

This study prospectively enrolled 304 APA patients who received adrenalectomy and 271 with essential hypertension (EH). Clinical, biochemical and echocardiographic data were collected in both groups and 1 year after surgery in the APA patients. The hemodynamic and non-hemodynamic components of left ventricular (LV) remodeling were represented by predicted left ventricular mass index (LVMI) (pLVMI) and inappropriately excessive LVMI (ieLVMI, defined as LVMI-pLVMI).

**Results:**

After propensity score matching, 213 APA and 213 EH patients were selected. APA patients had higher hemodynamic (pLVMI) and non-hemodynamic (ieLVMI) components of LV remodeling than EH patients. In multivariate analysis, baseline pLVMI was correlated with systolic blood pressure (SBP) and serum potassium, whereas ieLVMI was correlated with log plasma aldosterone concentration but not blood pressure. Post-operative echocardiography was available in 207 patents and showed significant decreases in both pLVMI and ieLVMI after adrenalectomy. In multivariate analysis, ΔpLVMI was correlated with SBP, ΔSBP, and pre-operative pLVMI, whereas ΔieLVMI was correlated with Δlog aldosterone-to-renin ratio (ARR) and pre-operative ieLVMI.

**Conclusions:**

This study concluded that extensive cardiac remodeling in APA patients occurs through hemodynamic and non-hemodynamic causes. Adrenalectomy can improve both hemodynamic and non-hemodynamic components of LV remodeling. Regressions of pLVMI and ieLVMI were correlated with decreases in blood pressure and ARR, respectively.

## Highlights

We investigated cardiac remodeling and reversal after adrenalectomy in 304 patients with aldosterone-producing adenoma (APA) with 271 essential hypertension (EH) through hemodynamic and non-hemodynamic components as predicted left ventricular mass index (LVMI) (pLVMI) and inappropriately excessive LVMI (ieLVMI). APA patients had higher pLVMI and ieLVMI correlated with blood pressure and aldosterone. 207 APA patents showed decreased pLVMI and ieLVMI after adrenalectomy with ΔpLVMI correlated with blood pressure and ΔieLVMI correlated with Δlog aldosterone-to-renin ratio. This study demonstrated improved cardiac remodeling in APA patients through hemodynamic and non-hemodynamic causes after adrenalectomy, which correlated with decreases in blood pressure and ARR, respectively.

## Introduction

Primary aldosteronism (PA) is a disease featuring excessive endogenous aldosterone (1, 2). Previous studies and a recent meta-analysis have reported an increased risk of cardiovascular diseases and greater cardiac remodeling in PA patients compared to those with essential hypertension (EH) (3, 4).

Cardiac remodeling is prognostic and linked to heart failure progression (5). It manifests clinically as changes in size, shape, and function of the heart, and is influenced by hemodynamic load, neurohormonal activation and other factors (5). Left ventricular hypertrophy (LVH) with increased cardiac mass is the most common and well-studied type of cardiac remodeling, and it has been clinically shown to be closely correlated to systolic and diastolic function, and to further predict long-term outcomes (6, 7).

Hypertension plays a key role in the hemostasis of hemodynamics, is a well-known risk factor for LVH (8), and is a major cause of LVH in patients with PA. However, PA with elevated blood pressure has been shown to cause a higher degree of LVH than hypertension itself, and an increased prevalence of LVH has been reported in patients with PA compared to those with EH after adjusting for blood pressure in previous retrospective cohorts (3). In addition, small prospective studies have demonstrated a higher incidence of LVH in PA patients than in blood pressure-matched EH controls (9). Excessive endogenous aldosterone has been reported to be another major cause of LVH, however these studies have included confounders such as the influence of blood pressure. Furthermore, the treatment of PA with unilateral adrenalectomy effectively decreases blood pressure and relieves aldosterone overproduction. Regression of LVH has been observed in previous studies in association with decreases in blood pressure and aldosterone or aldosterone-to-renin ratio (ARR) (9–11), however which component contributes most has yet to be clarified.

Inappropriate left ventricular mass is defined as the ratio between measured and predicted left ventricular mass taking sex, body size, and cardiac workload into account. It represents a non-hemodynamic or neurohormonal cause of LVH, and it has been reported to offer additional prognostic value in patients with LVH (12, 13). A previous study reported different distributions of inappropriate left ventricular mass among PA and EH groups (14). Inappropriately excessive left ventricular mass as a novel and promising parameter, the difference between measured and predicted left ventricular mass, may provide further information.

In this study, we compared measured, predicted, and inappropriately excessive left ventricular mass between a prospective cohort of patients with aldosterone-producing adenoma (APA) and EH controls, and evaluated their associations with cardiac remodeling and treatment response after unilateral adrenalectomy. We further investigated the pathogenesis of LVH due to hemodynamic and non-hemodynamic causes.

## Materials and Methods

### Participant Enrollment, Physiological and Laboratory Measurements

We enrolled patients with PA and EH from October 2006 to January 2016 from National Taiwan University Hospital. The PA patients were all registered in the Taiwan Primary Aldosteronism Investigation (TAIPAI) database (15). Detailed medical histories were collected from every patient including demographic characteristics and medications. EH was diagnosed according to standard algorithms after a thorough survey of the medical history and laboratory tests to exclude possible secondary hypertension. Comprehensive evaluations including physiological and laboratory studies and echocardiography were performed on enrollment (baseline), and follow-up evaluations were performed 12 months later after adrenalectomy in the APA patients. Clinical outcome of APA patients was classified as clinical cure and clinical non-cure. Clinical Cure refers to clinical complete success with normalization of BP with no antihypertensive medication use which is identical to the definition of “completely clinically cured” in the Primary Aldosteronism Surgical Outcomes (PASO) Classification System (16). Clinical non-cure refers clinical partial success and absent according to Primary Aldosteronism Surgical Outcome (PASO) Classification System (16).

Physiological assessments of systolic blood pressure (SBP) and diastolic blood pressure (DBP) were obtained using a sphygmomanometer according to clinical guidelines. Plasma aldosterone concentration (PAC) was measured using a radioimmunoassay with a commercial kit (Aldosterone Maia Kit; Adaltis Italia S.P.A., Bologna, Italy), and plasma renin activity (PRA) was measured as the generation of angiotensin-I *in vitro* using a commercially available radioimmunoassay kit (Cisbio, Bedford, Massachusetts, USA). All antihypertensive medications were discontinued for at least 21 days before measuring plasma PRA and PAC as suggested in clinical guidelines. Diltiazem and/or doxazosin were administered to lower elevated blood pressure when clinically indicated.

This study was conducted in compliance with the Declaration of Helsinki, and it was approved by the Institutional Review Board of National Taiwan University Hospital (Taipei, Taiwan). Informed consent was obtained from all individuals before enrollment.

### Diagnostic Criteria for Primary Aldosteronism and Subtype Identification

The confirmation and diagnosis of PA and further identification of APA were made according to previously published protocols and algorithms (2, 17). Patients who met the following three criteria were defined as having PA: (1) autonomous excess aldosterone production with an ARR > 35; (2) a TAIPAI score > 60%; and (3) post-saline loading PAC > 10 ng/dl, or PAC/PRA > 35 (ng/dl)/(ng/ml per h) in a post-captopril test, or PAC > 6 ng/dl in a fludrocortisone suppression test (2). APA was diagnosed in patients with PA and at least one of the following three conditions: (1) adenoma on a computed tomography (CT) scan for preoperative evaluation; (2) lateralization of aldosterone secretion evidenced by adrenal vein sampling or dexamethasone suppression NP-59 single photon emission computed tomography (SPECT)/CT; and (3) pathologically proven adenoma after surgery if the patients received an operation (2). The choice of unilateral adrenalectomy or medical treatment with mineralocorticoid receptor antagonists was discussed with the APA patients, along with a pre-operational assessment. The PA patients were enrolled after consenting to the surgery and if they were physically suitable for adrenalectomy.

### Echocardiographic Evaluation and Left Ventricle Geometry Calculation

A standardized echocardiographic ultrasound system (SONOS 5500 HP-Philips or IE33, Philips, Andover, Massachusetts, USA) was used, and transthoracic echocardiographic images were obtained in fundamental imaging modes by experienced and qualified technicians blind to the clinical diagnosis. M-mode measurements, two-dimensional imaging with standard views, and Doppler ultrasonography were acquired in each patient.

Left ventricular (LV) dimensions, septal and posterior wall thickness, and LVEF (M-mode) were measured *via* the parasternal long axis view according to the guidelines of the American Society of Echocardiography (18). Echocardiographic measured LV mass index (LVMI) was calculated according to the method of Devereux and Reichek: [LV mass = 1.04 x [(septal thickness + LV end-diastolic diameter + posterior wall thickness)^3^ - (LV end-diastolic diameter)^3^]-13.6] (19), then indexed with body mass index. Predicted LVMI (pLVMI) was estimated using a previously derived equation: predicted LVM = 55.37 + 6.64 x height^2.7^ + 0.64 x stroke work - 18.07 x gender (where gender was scored as male=1 and female=2) (20). Left ventricle volume was calculated using Tericholz’s formula, and stroke work was calculated as SBP (in mmHg) x stroke volume x 0.0144 (14). Inappropriately excessive LVMI (ieLVMI) was defined as: measured LVMI – predicted LVMI (21). LVH was defined according to Devereux’s criteria: LVMI ≥ 134 g/m2 in men and 110 g/m2 in women (22). The cut-off point of increased relative wall thickness (RWT) was set as 0.42, and LV morphology was classified (18). Concentric hypertrophy was defined as the presence of LVH and increased RWT; eccentric hypertrophy was defined as the presence of LVH without increased RWT; concentric remodeling was defined as the absence of LVH with increased RWT; and normal geometry was defined as the absence of LVH without increased RWT (18, 22). In this study, “Δ” denotes the difference between pre-operative and post-operative measurements calculated as the post-operative value – pre-operative value.

### Statistical Analysis

All continuous variables were expressed as mean ± SD if normally distributed and as median with interquartile range if non-normally distributed. The PA group and EH controls were matched using propensity score matching adjusted for age, sex, SBP, DBP, number of antihypertensive medication types, hypertension history using the Python-based extensions, FUZZY and PSM, in SPSS. All continuous variables were compared across groups using the Student’s t test (normally distributed) or the Wilcoxon rank-sum test (non-normally distributed), while the paired sample t-test was used if two groups were dependent. Categorical variables were presented as counts and percentages and were compared using the McNemar test. Equality of two proportions was assessed using the Pearson chi-square test. Data of PAC, PRA, and ARR were log-transformed due to non-normality as assessed using the Kolmogorov–Smirnov test. Pearson’s correlation tests were performed to determine correlations between LVMI/pLVMI/ieLVMI and ΔLVMI/ΔpLVMI/ΔieLVMI and clinical parameters. Significant determinants found in the Pearson’s correlation test (p ≦ 0.05) were then examined using a multivariate linear regression test with backward subset selection to identify independent factors predicting LVMI/pLVMI/ieLVMI and ΔLVMI/ΔpLVMI/ΔieLVMI. All statistical analyses were performed using SPSS for Windows version 25.0 (SPSS Inc., Chicago, Illinois, USA). All tests were two-tailed, and a p value of ≤ 0.05 was considered to indicate statistical significance.

## Results

### Patient Characteristics and Demographics

In this study, we enrolled 304 patients with PA, all of whom had APA, and 271 EH patients. The basic clinical characteristics of the patients are listed in **Table 1**. More of the APA group were female. In addition, the APA group had significantly higher SBP and DBP, longer duration of hypertension, used more types of antihypertensive medications, and had lower serum creatinine and potassium levels than the EH group. Moreover, the APA group had significantly higher serum PAC, lower PRA, and higher derived ARR than the EH group, and the differences remained after log-transformation. Other parameters were comparable between the two groups. The APA group used more antihypertensive medications compared to the EH controls, except for vasodilators and diuretics. After propensity-score matching adjusting for age, sex, SBP and DBP, duration of hypertension, and total number of antihypertensive types, we successfully matched 213 APA patients to 213 EH patient (**Table 1**). There were no significant differences among the clinical characteristics of the two groups except for lower PRA, log-transformed PRA, serum potassium and creatinine levels, and higher PAC, log-transformed PAC, ARR, and log-transformed ARR in the APA group. More of the EH group used angiotensin receptor blockers and more of the APA group used alpha-blockers, however there were no significant differences in other antihypertensive medications between the two groups.

**Table 1:** Clinical characteristics of patients with primary aldosteronism and essential hypertension.

Unmatched data				Propensity-score matching			
Patient characteristics	Primary aldosteronism(n = 304)	Essential hypertension(n = 271)	p value	Patient characteristics	Primary aldosteronism(n = 213)	Essential hypertension(n = 213)	p value
Sex (Male), n (%)	132 (43.4%)	146 (53.9%)	0.012	Sex (Male), n (%)	102 (47.9%)	107 (50.2%)	0.628
Age (years)	50.9 ± 11.3	52.7 ± 14.9	0.109	Age (years)	51.3 ± 11.2	52.0 ± 13.8	0.592
Body height (cm)	162.5 ± 8.5	163.5 ± 9.6	0.163	Body height (cm)	163.1 ± 8.4	163.0 ± 9.9	0.935
Body weight (kg)	67.4 ± 14.0	69.2 ± 14.9	0.152	Body weight (kg)	67.8 ± 13.8	68.9 ± 15.2	0.417
BMI (kg/m^2^)	25.4 ± 4.0	25.7 ± 4.3	0.340	BMI (kg/m^2^)	25.3 ± 3.8	25.8 ± 4.3	0.258
BSA (m^2^)	1.72 ± 0.20	1.75 ± 0.22	0.063	BSA (m^2^)	1.73 ± 0.20	1.74 ± 0.22	0.429
HR (bpm)	73.0 ± 12.6	74.0 ± 13.6	0.399	HR (bpm)	72.8 ± 11.9	73.8 ± 13.2	0.448
SBP (mmHg)	154.1 ± 20.3	146.0 ± 21.7	< 0.001	SBP (mmHg)	149.7 ± 18.3	147.6 ± 21.7	0.279
DBP (mmHg)	91.8 ± 13.5	85.7 ± 13.7	< 0.001	DBP (mmHg)	88.5 ± 11.9	86.9 ± 13.5	0.201
Serum creatinine level (mg/dl)	0.92 ± 0.40	1.03 ± 0.57	0.008	Serum creatinine level (mg/dl)	0.91 ± 0.39	1.01 ± 0.58	0.044
Serum potassium level (mmol/dl)	3.52 ± 0.68	4.17 ± 0.41	< 0.001	Serum potassium level (mmol/dl)	3.56 ± 0.68	4.14 ± 0.41	< 0.001
PAC (ng/dl)^a^	44.92 (42.38)	32.46 (26.93)	< 0.001	PAC (ng/dl)^a^	45.18 (42.40)	31.74 (25.20)	< 0.001
PRA (ng/ml per h)^a^	0.21 (0.49)	2.00 (4.66)	< 0.001	PRA (ng/ml per h)^a^	0.21 (0.50)	2.17 (5.30)	< 0.001
ARR^a^	229.15 (745.14)	15.74 (39.65)	< 0.001	ARR^a^	222.00 (741.50)	15.34 (33.00)	< 0.001
Log-transformed PAC	1.68 ± 0.26	1.51 ± 0.25	< 0.001	Log-transformed PAC	1.67 ± 0.26	1.51 ± 0.25	< 0.001
Log-transformed PRA	-0.75 ± 0.71	0.25 ± 0.71	< 0.001	Log-transformed PRA	-0.75 ± 0.69	0.28 ± 0.70	< 0.001
Log-transformed ARR	2.43 ± 0.76	1.25 ± 0.72	< 0.001	Log-transformed ARR	2.42 ± 0.74	1.21 ± 0.72	< 0.001
Number of antihypertensive medication type	2.2 ± 1.3	1.8 ± 1.1	< 0.001	Number of antihypertensive medication type	2.0 ± 1.2	1.9 ± 1.1	0.643
Hypertension history (years)	8.0 ± 7.4	6.4 ± 7.8	0.016	Hypertension history (years)	7.1 ± 6.7	7.2 ± 8.1	0.897
Hypertension medication				Hypertension medication			
ACEI, n (%)	14 (4.6%)	4 (1.5%)	0.031	ACEI, n (%)	10 (4.7%)	4 (1.9%)	0.103
ARB, n (%)	119 (39.1%)	145 (53.5%)	0.001	ARB, n (%)	73 (34.3%)	130 (61.0%)	< 0.001
Alpha-blocker, n (%)	74 (24.3%)	27 (10.0%)	< 0.001	Alpha-blocker, n (%)	48 (22.5%)	25 (11.7%)	0.003
Beta-blocker, n (%)	120 (39.5%)	81 (29.9%)	0.016	Beta-blocker, n (%)	71 (33.3%)	63 (29.6%)	0.404
CCB, n (%)	217 (71.4%)	167 (61.6%)	0.013	CCB, n (%)	146 (68.5%)	142 (66.7%)	0.679
Vasodilator, n (%)	18 (5.9%)	15 (5.5%)	0.843	Vasodilator, n (%)	9 (4.2%)	13 (6.1%)	0.381
Diuretics, n (%)	31 (10.2%)	26 (9.6%)	0.809	Diuretics, n (%)	19 (8.9%)	24 (11.3%)	0.421

Values are expressed as mean ± SD, median (interquartile range), or number (percentage).

ACEI, angiotensin-converting enzyme inhibitor; ARB, AT1 blocker; ARR, aldosterone–renin ratio; CCB, calcium channel blocker; PAC, plasma aldosterone concentration; PRA, plasma renin activity.

a Expressed as median and interquartile range.

### Echocardiographic Comparison Between APA and EH

**Figure 1:**
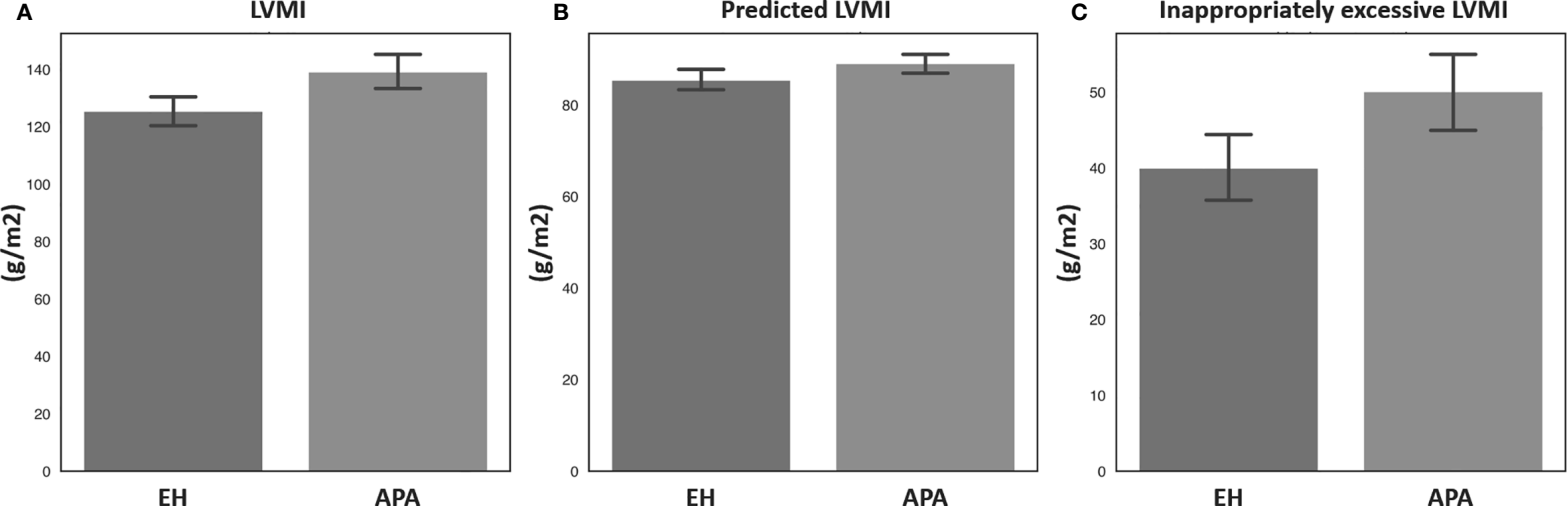
Comparisons of **(A)** LVMI, **(B)** predicted LVMI, and **(C)** inappropriately excessive LVMI between patients with aldosterone-producing adenoma and essential hypertension after propensity score matching. APA, aldosterone-producing adenoma; EH, essential hypertension; LVMI, left ventricular mass index.

Echocardiographic parameters were then compared between the APA and EH groups before and after propensity score matching (**Table 2**). Both before and after propensity score matching, the APA group had a significantly higher LVMI, pLVMI, and ieLVMI compared to the EH controls. In addition, the APA group had significantly higher percentages of LVH and concentric hypertrophy before and after matching. Comparisons of LVMI, pLVMI, and ieLVMI between the APA and EH groups after propensity-score matching are shown in **Figure 1**.

**Table 2:** Echocardiographic features and Doppler-derived indexes of patients with primary aldosteronism and essential hypertension.

Unmatched data				Propensity-score matching			
Echographic parameters	Primary aldosteronism (n = 304)	Essential hypertension (n = 271)	p value	Echographic parameters	Primary aldosteronism(n = 213)	Essential hypertension(n = 213)	p value
LVEDD (cm)	4.73 ± 0.48	4.64 ± 0.50	0.037	LVEDD (cm)	4.72 ± 0.49	4.66 ± 0.51	0.177
LVESD (cm)	2.84 ± 0.44	2.83 ± 0.52	0.801	LVESD (cm)	2.86 ± 0.45	2.81 ± 0.51	0.302
IVSD (cm)	1.19 ± 0.22	1.14 ± 0.23	0.012	IVSD (cm)	1.18 ± 0.22	1.14 ± 0.22	0.098
LVPWD (cm)	1.12 ± 0.18	1.08 ± 0.19	0.011	LVPWD (cm)	1.11 ± 0.18	1.07 ± 0.18	0.049
RWT	0.48 ± 0.08	0.47 ± 0.09	0.269	RWT	0.47 ± 0.08	0.46 ± 0.08	0.284
LVEF (%)	70.2 ± 7.6	69.2 ± 7.9	0.118	LVEF (%)	69.7 ± 8.0	69.7 ± 7.6	0.998
Transmitral E velocity (cm/s)	75.72 ± 19.44	76.82 ± 19.30	0.504	Transmitral E velocity (cm/s)	75.73 ± 19.42	76.88 ± 18.57	0.537
Transmitral A velocity (cm/s)	80.25 ± 18.06	80.72 ± 18.46	0.765	Transmitral A velocity (cm/s)	80.00 ± 17.96	80.43 ± 18.36	0.814
E deceleration time (ms)	212.7 ± 44.8	206.7 ± 52.9	0.159	E deceleration time (ms)	210.9 ± 42.8	206.0 ± 51.2	0.298
LVMI (g/m2)	142.04 ± 42.53	125.92 ± 39.00	<0.001	LVMI (g/m2)	139.17 ± 43.01	125.67 ± 36.32	0.001
Predicted LVMI (g/m2)	91.37 ± 17.37	84.22 ± 17.23	<0.001	Predicted LVMI (g/m2)	89.08 ± 15.77	85.47 ± 16.33	0.024
Inappropriately excessive LVMI (g/m2)	50.67 ± 35.89	42.09 ± 35.19	0.006	Inappropriately excessive LVMI (g/m2)	50.09 ± 35.97	39.99 ± 30.91	0.003
LVH, n (%)	199 (68.2%)	114 (44.7%)	<0.001	LVH, n (%)	130 (64.4%)	90 (44.1%)	<0.001
LV morphology, n (%)				LV morphology, n (%)			
Concentric hypertrophy, n (%)	165 (56.5%)	95 (37.3%)	<0.001	Concentric hypertrophy, n (%)	109 (54.0%)	72 (35.3%)	<0.001
Eccentric hypertrophy,n (%)	34 (11.6%)	19 (7.5%)	0.098	Eccentric hypertrophy, n (%)	21 (10.4%)	18 (8.8%)	0.591
Concentric remodeling, n (%)	60 (20.5%)	85 (33.3%)	0.001	Concentric remodeling, n (%)	45 (22.3%)	67 (32.8%)	0.017
Normal geometry, n (%)	33 (11.3%)	56 (22.0%)	0.001	Normal geometry, n (%)	27 (13.4%)	47 (23.0%)	0.012

Values are expressed as mean ± SD or number (percentage). IVSD, interventricular septal end diastole thickness; LAD, left atrial diameter; LADI, left atrial diameter index adjusted for BSA; LVEDD, left ventricular end-diastolic diameter; LVEF, left ventricular ejection fraction; LVESD, left ventricular end-systolic diameter; LVH, left ventricular hypertrophy; LVM, left ventricular mass; LVMI, left ventricular mass index; LVPWD, left ventricular posterior wall end diastole thickness; RWT, relative wall thickness.

### Factors Affecting LVMI/pLVMI/ieLVMI in the APA and EH Groups

Regression analysis of factors predicting LVMI/pLVMI/ieLVMI in the study cohort are listed in **Table 3**. In multivariable analysis, sex (β = - 0.158, p = 0.001), SBP (β = 0.151, p = 0.001), serum creatinine level (β = 0.199, p < 0.001), serum potassium level (β = -0.133, p = 0.005), log-transformed PAC (β = 0.141, p = 0.003), and number of antihypertensive medication types (β = 0.134, p = 0.006) were independently associated with LVMI. In addition, sex (β = - 0.270, p < 0.001), SBP (β = 0.644, p < 0.001), DBP (β = -0.256, p < 0.001) and potassium level (β = -0.158, p < 0.001) were independently associated with pLVMI, and the presence of PA (β = 0.131, p = 0.008), body mass index (β = 0.143, p = 0.002), serum creatinine level (β = 0.240, p < 0.001), log-transformed PAC (β = 0.123, p = 0.012), and number of antihypertensive medication types (β = 0.145, p = 0.003) were independently associated with ieLVMI.

**Table 3:** Correlation study between LVMI, pLVMI, and ieLVMI and clinical parameters (after propensity-score matching adjusted for age, sex, SBP, DBP, antihypertensive types, hypertension years) (n = 426).

Variable	LVMI				pLVMI				ieLVMI			
	Univariate regression		Multivariate regression		Univariate regression		Multivariate regression		Univariate regression		Multivariate regression	
	β (95% C.I)	p value	β (95% C.I)	p value	β (95% C.I)	p value	β (95% C.I)	p value	β (95% C.I)	p value	β (95% C.I)	p value
Presence of PA	0.168 (0.071, 0.264)	0.001			0.112 (0.015, 0.209)	0.024			0.149 (0.053, 0.246)	0.003	0.131 (0.035, 0.227)	0.008
Sex (male)	-0.262 (-0.356, -0.168)	< 0.001	-0.158 (-0.252, -0.063)	0.001	-0.300 (-0.393, -0.206)	< 0.001	-0.270 (-0.350, -0.190)	< 0.001	-0.168 (-0.264, -0.071)	0.001		
Age	0.023 (-0.074, 0.121)	0.640			0.025 (-0.073, 0.123)	0.620			0.019 (-0.079, 0.117)	0.698		
BMI	0.095 (-0.002, 0.191)	0.057			-0.069 (-0.167, 0.028)	0.164			0.144 (0.047, 0.241)	0.004	0.143 (0.051, 0.236)	0.002
SBP	0.285 (0.191, 0.379)	< 0.001	0.151 (0.059, 0.241)	0.001	0.545 (0.463, 0.627)	< 0.001	0.644 (0.539, 0.750)	< 0.001	0.083 (-0.014, 0.181)	0.094		
DBP	0.080 (-0.017, 0.177)	0.109			0.253 (0.158, 0.348)	< 0.001	-0.256 (-0.363, -0.149)	< 0.001	-0.024 (-0.122, 0.074)	0.627		
Serum creatinine level	0.284 (0.189, 0.377)	< 0.001	0.199 (0.100, 0.296)	< 0.001	0.162 (0.065, 0.259)	0.001			0.261 (0.167, 0.356)	< 0.001	0.240 (0.144, 0.337)	< 0.001
Serum potassium level	-0.183 (-0.279, -0.086)	< 0.001	-0.133 (-0.225, -0.041)	0.005	-0.204 (-0.300, -0.108)	<0.001	-0.158 (-0.237, -0.079)	< 0.001	-0.123 (-0.220, -0.025)	0.014		
Log-transformed PAC	0.163 (0.066, 0.260)	0.001	0.141 (0.049, 0.232)	0.003	0.101 (0.003, 0.199)	0.043			0.141 (0.044, 0.238)	0.005	0.123 (0.027, 0.218)	0.012
Log-transformed PRA	-0.130 (-0.227, -0.032)	0.009			-0.101 (-0.199, -0.003)	0.043			-0.107 (-0.205, -0.009)	0.032		
Log-transformed ARR	0.142 (0.044, 0.238)	0.004			0.104 (0.007, 0.202)	0.036			0.120 (0.022, 0.217)	0.016		
Number of antihypertensive medication type	0.284 (0.190, 0.378)	< 0.001	0.134 (0.039, 0.228)	0.006	0.173 (0.076, 0.269)	< 0.001			0.259 (0.164, 0.354)	< 0.001	0.145 (0.048, 0.243)	0.003
Hypertension history	0.157 (0.060, 0.253)	0.001			0.125 (0.028, 0.222)	0.012			0.123 (0.026, 0.220)	0.013		

ARR, aldosterone–renin ratio; LVMI, left ventricular mass index; PAC, plasma aldosterone concentration; PRA, plasma renin activity.

### Clinical and Echocardiographic Evaluations in the APA Patients Before and After Adrenalectomy

**Figure 2:**
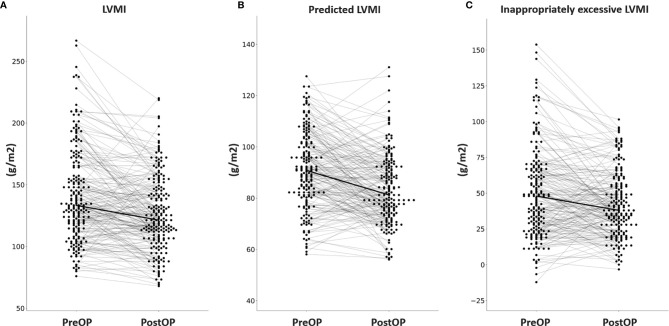
Changes in **(A)** LVMI, **(B)** predicted LVMI, **(C)** and inappropriately excessive LVMI in aldosterone-producing adenoma patients before and after adrenalectomy. LVMI, left ventricular mass index; PreOP, before adrenalectomy; PostOP, after adrenalectomy.

Among the 304 APA patients, echocardiography data 12 months after adrenalectomy were available in 207, and their clinical and echocardiographic data are listed in **Table 4**. Significantly lower SBP and DBP, and significantly higher serum potassium and creatinine levels were noted after adrenalectomy. In addition, a significantly lower number of antihypertensive types was observed, along with significant decreases in PAC, log-transformed PAC, ARR, and log-transformed ARR, and significant increases in PRA and log-transformed PRA after adrenalectomy. Interventricular septal diameter, left ventricular posterior wall diameter and RWT decreased significantly after adrenalectomy, whereas left ventricular end-diastolic diameter, left ventricular end-systolic diameter and left ventricular ejection fraction did not. There were also significant decreases in LVMI, pLVMI, and ieLVMI after adrenalectomy. Changes in LVMI, pLVMI and ieLVMI in the APA patients before and after adrenalectomy are shown in **Figure 2**. The percentage of LVH decreased significantly after adrenalectomy.

**Table 4:** Clinical characteristics, echocardiographic features, doppler-derived indexes, and change of patients with primary aldosteronism receiving adrenalectomy.

Patient characteristics	Pre-OP	Post-OP	p value
SBP (mmHg)	154.8 ± 20.1	137.7 ± 18.7	<0.001
DBP (mmHg)	92.3 ± 13.9	84.8 ± 11.6	<0.001
Serum creatinine level (mg/dl)	0.91 ± 0.38	1.06 ± 0.66	<0.001
Serum potassium level (mmol/dl)	3.60 ± 0.68	4.32 ± 0.54	<0.001
PAC (ng/dl)^a^	45.60 (43.60)	28.55 (22.00)	<0.001
PRA (ng/ml per h)^a^	0.23 (0.50)	1.76 (4.20)	<0.001
ARR^a^	234.29 (1107.8)	16.88 (35.70)	<0.001
Log-transformed PAC	1.71 ± 0.25	1.47 ± 0.27	<0.001
Log-transformed PRA	-0.76 ± 0.74	0.18 ± 0.63	<0.001
Log-transformed ARR	2.46 ± 0.78	1.29 ± 0.64	<0.001
Number of antihypertensive medication type	2.3 ± 1.3	0.7 ± 1.1	<0.001
Echocardiographic variables	Pre-OP	Post-OP	p value
LVEDD (cm)	4.74 ± 0.46	4.71 ± 0.46	0.186
LVESD (cm)	2.84 ± 0.42	2.83 ± 0.42	0.414
IVSD (cm)	1.19 ± 0.21	1.12 ± 0.19	<0.001
LVPWD (cm)	1.13 ± 0.18	1.05 ± 0.15	<0.001
RWT	0.48 ± 0.08	0.45 ± 0.08	<0.001
LVEF (%)	70.2 ± 7.1	70.1 ± 7.0	0.867
Transmitral E velocity (cm/s)	75.2 ± 18.8	73.8 ± 17.5	0.256
Transmitral A velocity (cm/s)	80.5 ± 17.6	79.5 ± 18.3	0.364
E deceleration time (ms)	212.7 ± 44.9	223.0 ± 71.2	0.059
LVMI (g/m2)	142.07 ± 40.60	127.39 ± 34.61	<0.001
Predicted LVMI (g/m2)	90.89 ± 16.23	83.89 ± 16.19	<0.001
Inappropriately excessive LVMI (g/m2)	50.89 ± 33.86	42.90 ± 26.92	<0.001
LVH, n (%) (n = 207)	140 (67.6%)	116 (56.0%)	0.002

Values are expressed as mean ± SD, median (interquartile range), or number (percentage).

ARR, aldosterone–renin ratio; PAC, plasma aldosterone concentration; PRA, plasma renin activity.

a Expressed as median and interquartile range. (n = 207).

Comparison of the changes before and after operation of APA according to clinical cure or clinical non-cure was demonstrated in **Supplements Table 1** and **2**. Patients in clinical cure group had less duration of hypertension and greater antihypertensive medication reduction after adrenalectomy than patients in clinical non-cure group. Patients in both groups had significant reduction of SBP, DBP, PAC, and ARR while patients in clinical cure group had more reduction than patients in clinical non-cure group. Patients in both groups had significant reduction of LVMI pLVMI and ieLVMI expect pLVMI in clinical non-cure group which is borderline significant (p=0.058). The difference of ΔLVMI between the two groups is borderline significant (p = 0.086). Patients in clinical cure group had significantly higher ΔpLVMI than non-cure group (p<0.001). The difference of ΔieLVMI between the two groups is not significant (p = 0.450).

### Factors Affecting Changes in LVMI/pLVMI/ieLVMI in the APA Patients

Regression analysis of factors predicting changes in LVMI/pLVMI/ieLVMI after adrenalectomy among the APA group are listed in **Table 5**. In multivariable analysis, SBP (β = -0.244, p = 0.001), ΔSBP (β = 0.268, p < 0.001), Δlog-transformed ARR (β = 0.199, p < 0.001) and pre-operative LVMI (β = 0.687, p < 0.001) were significantly associated with ΔLVMI; SBP (β = -0.411, p < 0.001), ΔSBP (β = 0.655, p < 0.001) and pre-operative pLVMI (β = 0.561, p < 0.001) were significantly associated with ΔpLVMI; and Δlog-transformed ARR (β = 0.155, p = 0.004) and pre-operative ieLVMI (β = 0.664, p < 0.001) were significantly associated with ΔieLVMI.

**Table 5:** Correlation study between ΔLVMI, ΔpLVMI, ΔieLVMI and clinical parameters (unmatched).

Variable	ΔLVMI				ΔpLVMI				ΔieLVMI			
	Univariate regression		Multivariate regression		Univariate regression		Multivariate regression		Univariate regression		Multivariate regression	
	β (95% C.I)	p value	β (95% C.I)	p value	β (95% C.I)	p value	β (95% C.I)	p value	β (95% C.I)	p value	β (95% C.I)	p value
Sex (male)	-0.036 (-0.173, 0.102)	0.611			-0.102 (-0.243, 0.039)	0.155			-0.033 (-0.175, 0.109)	0.648		
Age	-0.150 (-0.286, -0.014)	0.031			-0.101 (-0.242, 0.041)	0.162			-0.066 (-0.208, 0.075)	0.356		
BMI	0.024 (-0.113, 0.161)	0.733			0.015 (-0.127, 0.157)	0.835			0.045 (-0.097, 0.187)	0.531		
SBP	0.177 (0.041, 0.312)	0.011	-0.244 (-0.390, -0.099)	0.001	0.350 (0.217, 0.483)	< 0.001	-0.411 (-0.562, -0.260)	< 0.001	-0.008 (-0.150, 0.134)	0.913		
ΔSBP	0.251 (0.113, 0.388)	< 0.001	0.268 (0.129, 0.406)	< 0.001	0.613 (0.501, 0.725)	< 0.001	0.655 (0.530, 0.780)	< 0.001	-0.077 (-0.219, 0.064)	0.282		
DBP	0.117 (-0.019, 0.254)	0.092			0.209 (0.070, 0.348)	0.003			-0.004 (-0.146, 0.138)	0.955		
ΔDBP	0.153 (0.012, 0.293)	0.033			0.370 (0.238, 0.502)	< 0.001			-0.045 (-0.187, 0.097)	0.535		
Serum creatinine level	-0.068 (-0.205, 0.069)	0.332			0.029 (-0.113, 0.171)	0.690			-0.031 (-0.173, 0.111)	0.667		
ΔSerum creatinine level	-0.136 (-0.278, 0.006)	0.062			-0.193 (-0.336, 0.050)	0.009			-0.036 (-0.182, 0.110)	0.625		
Serum potassium level	-0.176 (-0.311, -0.040)	0.011			-0.219 (-0.358, -0.081)	0.002			-0.119 (-0.260, 0.022)	0.097		
ΔSerum potassium level	-0.187 (-0.326, -0.047)	0.009			-0.206 (-0.346, -0.065)	0.004			-0.106 (-0.249, 0.037)	0.144		
Log-transformed PAC	0.165 (0.029, 0.300)	0.018			0.126 (-0.015, 0.267)	0.079			0.083 (-0.059, 0.224)	0.251		
ΔLog-transformed PAC	0.205 (0.065, 0.344)	0.004			0.187 (0.045, 0.329)	0.010			0.127 (-0.016, 0.271)	0.082		
Log-transformed PRA	-0.073 (-0.210, 0.063)	0.293			-0.067 (-0.208, 0.075)	0.355			-0.060 (-0.201, 0.082)	0.407		
ΔLog-transformed PRA	-0.189 (-0.329, -0.049)	0.008			-0.168 (-0.311, -0.025)	0.021			-0.117 (-0.261, 0.027)	0.111		
Log-transformed ARR	0.121 (-0.015, 0.257)	0.082			0.103 (-0.038, 0.244)	0.151			0.083 (-0.059, 0.224)	0.250		
ΔLog-transformed ARR	0.288 (0.150, 0.425)	< 0.001	0.199 (0.092, 0.306)	< 0.001	0.242 (0.100, 0.383)	0.001			0.182 (0.038, 0.325)	0.013	0.155 (0.049, 0.261)	0.004
Number of antihypertensive medication type	0.019 (-0.118, 0.156)	0.786			0.036 (-0.105, 0.178)	0.613			0.002 (-0.140, 0.143)	0.983		
Change of number of antihypertensive medication type	0.038 (-0.102, 0.178)	0.598			0.076 (-0.068, 0.221)	0.298			0.012 (-0.133, 0.157)	0.869		
Hypertension history	-0.025 (-0.162, 0.112)	0.722			0.047 (-0.095, 0.189)	0.513			-0.048 (-0.189, 0.094)	0.509		
Clinical success to off medication	0.078 (-0.061, 0.218)	0.272			0.106 (-0.038, 0.250)	0.148			0.055 (-0.090, 0.200)	0.454		
pre-OP LVMI	0.577 (0.465, 0.690)	< 0.001	0.687 (0.574, 0.800)	< 0.001	0.254 (0.116, 0.391)	< 0.001			0.538 (0.418, 0.657)	< 0.001		
pre-OP pLVMI	0.283 (0.151, 0.416)	< 0.001			0.515 (0.393, 0.637)	< 0.001	0.561 (0.433, 0.689)	< 0.001	0.048 (-0.094, 0.190)	0.505		
pre-OP ieLVMI	0.555 (0.441, 0.670)	< 0.001			0.061 (-0.081, 0.202)	0.401			0.628 (0.518, 0.739)	< 0.001	0.664 (0.558, 0.771)	< 0.001

Correlation analysis, univariate, and multivariate linear regression analysis for changes in LVMI among total PA patients (n = 207).

ARR, aldosterone–renin ratio; LVMI, left ventricular mass index; PAC, plasma aldosterone concentration; PRA, plasma renin activity.

## Discussion

There are five findings in this study. First, after propensity score matching for age, sex, SBP, DBP, hypertension duration, and use of antihypertensive, the APA patients had greater LVMI, pLVMI, and ieLVMI, with more LVH compared to the EH patients. Second, LVMI was independently associated with SBP, number of antihypertensive medication types, and log-transformed PAC, while pLVMI (hemodynamic component of LV remodeling) was associated with SBP, and ieLVMI was associated with log-transformed PAC (non-hemodynamic component of LV remodeling). Third, significant regressions of LVMI, pLVMI and ieLVMI and reversal of LVH were observed after adrenalectomy. Fourth, ΔLVMI was associated with SBP, ΔSBP, and Δlog-transformed ARR, while ΔpLVMI was associated with SBP, and ΔSBP and ΔieLVMI were associated with Δlog-transformed ARR. These findings clearly showed the factors associated with hemodynamic and non-hemodynamic component of LV remodeling in PA patients, and the factors associated with the regression of both components after adrenalectomy. Last, the study demonstrates that inappropriately excessive left ventricular mass (ieLVM) could be a novel and promising parameter evaluating aldosterone-induced non-hemodynamic left ventricular remodeling.

A previous meta-analysis reported that LVH identified by ECG or echocardiography was highly prognostic and an independent risk factor for cardiovascular outcomes (23). In addition, regression of LVH after antihypertensive medication has been associated with a significant reduction in cardiovascular risk compared with persistent or new-onset LVH (24). Besides being an initial step in clinical diseases, LV hypertrophy has also been proposed to be a compensatory process for abnormal loading conditions in studies taking pLVMI with gender, cardiac loading condition, and body size into consideration (8, 20, 23, 25). Blood pressure has also been shown to be a central factor for the onset and progression of LVH (7), however, many nonhemodynamic factors have also been implicated in the pathogenesis of hypertensive LVH (26). For example, LVMI was related to plasma fibrinogen and aldosterone in EH patients after adjusting age, blood pressure, and body mass index (27). In another study, post-saline load plasma aldosterone is positively related to left ventricular mass independent of blood pressure (28).

An inappropriate increase in LVM, which is referred to as an abnormal (non-compensatory) increase in LVM as opposed to a normal (compensatory) LVM increase, has been reported to be more strongly associated with more metabolic risk factors (29). Previous studies have reported a higher cardiovascular risk in individuals with inappropriate LVM even without traditionally defined LVH (12, 13, 30), and the additional prognostic value of changes in inappropriate LVM has been observed in those with traditionally defined LVH (12, 13). Inappropriate LVM is thus an ideal method to evaluate nonhemodynamic or neurohumoral factors in the pathogenesis of LVH in patients with PA after eliminating the influence of sex, body size, and cardiac workload which is increased in PA because of elevated blood pressure. Muiesan et al. in 2008 were the first to report a prospective cross-sectional study comparing PA patients with EH controls to assess the non-hemodynamic effect of excessive aldosterone on LVM (14). They found a higher prevalence of inappropriate LVM among PA patients with traditionally defined LVH compared with EH controls, and a trend of a higher prevalence of inappropriate LVM among PA patients without traditionally defined LVH. These findings provided evidence of an aldosterone-induced increase in LVM exceeding the amount needed to compensate for hemodynamic load.

Several previous studies have reported differences in LVMI and LVH between PA and EH groups and improvements after surgery or medications. Rossi et al. reported increased LV wall thickness and LVMI with higher percentages of LVH and concentric remodeling in PA patients compared to matched EH controls, and that adrenalectomy markedly reduced LV wall thickness and LVMI in APA patients (9). Catena et al. reported a greater LVM and more LVH among PA patients compared to EH controls with improvements after treatment with adrenalectomy or spironolactone (31). In our previous study of 30 APA patients, we also found a decreased LVMI in patients with LVH after adrenalectomy, and a decreased LVMI was correlated with preoperative LVMI, and postoperative changes in SBP and potassium level (10, 11, 32). We also found significant correlations among 24-hour urinary aldosterone, sodium, glomerular hyperfiltration and LVMI and ieLVMI among PA patients, as well as diastolic dysfunction (21, 33–35).

To the best of our knowledge, this prospective cohort study includes the largest sample size to date, with 304 PA patients (all of whom had APA) and 271 EH controls, and 213 matched pairs. The cases were matched using propensity score matching for sex, age, SBP, DBP, number of antihypertensive medication types, and hypertension history, with the aim of eliminating as many confounding factors as possible. The use of ieLVMI is different from previous studies and demonstrated the effect of excessive aldosterone on LVH in addition to adaptation to compensate for hemodynamic load. Our findings may help to improve the prediction of cardiovascular outcomes after eliminating the influence of hypertension. The utilization of LVMI, pLVMI and ieLVMI provided further insight into the pathogenesis of increased LVMI, a process involving mixed etiologies, in which pLVMI represented the hemodynamic component and ieLVMI the non-hemodynamic component. Correlation studies of LVMI/pLVMI/ieLVMI between the patients with APA and EH identified the factors associated with increased LVMI, while correlation studies of ΔLVMI/ΔpLVMI/ΔieLVMI after adrenalectomy identified the factors associated with changes in LV morphology and treatment effect of adrenalectomy. Of note, ΔLVMI was independently correlated with ΔSBP and ΔlogARR. Furthermore, ΔpLVMI was independently correlated with ΔSBP, while ΔieLVMI was correlated with ΔlogARR. These findings emphasize the treatment benefits of adrenalectomy, which both reduces blood pressure and aldosterone by removing the aldosterone secreting source in the adrenal gland, thereby resulting in LVH regression in both hemodynamic and non-hemodynamic pathways. The use of ieLVMI has the advantage of excluding the influence of blood pressure on the left structure. In addition, the exclusive association between ΔieLVMI and ΔlogARR consolidates the pathogenesis of aldosterone-induced neurohormonal LVH, which is independent of the influence of hypertension. Moreover, this shows that the treatment effect of adrenalectomy was not just from treating hypertension.

## Possible Future Study and Study Limitations

There are several limitations to this study. First, this clinical study showed the association between preoperative and postoperative endocrinological and cardiac structural changes, however the study design cannot elucidate the actual causative role of aldosterone in LV remodeling. Second, our follow-up period was 1 year, and longer follow-up periods are warranted to examine whether there is any further late-onset effect of adrenalectomy. Third, we did not perform the saline load test with plasma aldosterone measurement and fibrinogen level on all the APA patients before and after the operation. Fourth, this study only investigated excess aldosterone and showed that elevated ARR increased LVMI, but the effect on cardiovascular mortality or morbidity was not investigated. Further long-term follow-up studies are needed to investigate the clinical impact of aldosterone excess on cardiovascular outcomes.

## Conclusion

Extensive cardiac remodeling through hemodynamic and non-hemodynamic causes occurs in APA patients. Adrenalectomy improved both hemodynamic and non-hemodynamic components of LV remodeling. The regression of pLVMI and ieLVMI were correlated with decreases in blood pressure and ARR, respectively. These findings provide further evidence of aldosterone-induced hemodynamic and non-hemodynamic LV remodeling in patients with PA, and the effect of adrenalectomy.

## Data Availability Statement

The raw data supporting the conclusions of this article will be made available by the authors, without undue reservation.

## Ethics Statement

The studies involving human participants were reviewed and approved by National Taiwan University Hospital (NTUH) Research Ethics Committee (REC). The patients/participants provided their written informed consent to participate in this study.

## Author Contributions

C-TP: experimental design and manuscript writing. X-MW, C-HT, Y-YC and ZW-C: patient enrollment and experimental design. C-CC and B-CL: diagnosis helping, AVS, and report. C-WL and Y-LC: patient case management and experimental design. L-CL and Y-RC: diagnosis helping, echocardiography, experimental design. C-SH and Y-HL: patient enrollment, experimental design, funding raising, manuscript editing. All authors contributed to the article and approved the submitted version.

## Funding

This study was partially supported by grants from the Ministry of Science and Technology (MOST 106-2314-B-002 -048 –MY3) and National Taiwan University Hospital (NTUH 109-A141). The funders had no role in study design, data collection and analysis, decision to publish, or preparation of the manuscript.

## Conflict of Interest

The authors declare that the research was conducted in the absence of any commercial or financial relationships that could be construed as a potential conflict of interest.

## Abbreviations

PA, primary aldosteronism; EH, essential hypertension; APA, Aldosterone-producing adenoma; ARR, aldosterone-to-renin ratio; LVMI, left ventricular mass index; pLVMI, predicted left ventricular mass index; ieLVMI, inappropriately excessive left ventricular mass index.
