# Supplementary material for: Hemodynamic and Non-Hemodynamic Components of Cardiac Remodeling in Primary Aldosteronism
Source: Front Endocrinol (Lausanne). 2021 Apr 19;12:646097. doi: 10.3389/fendo.2021.646097 (PMC8092478; doi:10.3389/fendo.2021.646097)
Supplement: Supplementary file 1 [file Table_1.docx]

| **Supplement Table 1. Pre- and post- OP clinical characteristics and echographic parameters of APA patients with different clinical outcome.** | | | | | | | | | | |
| --- | --- | --- | --- | --- | --- | --- | --- | --- | --- | --- |
| Patient characteristics | | | | Clinical cure (n = 95) | | | Clinical non-cure (n = 112) | | p value | |
| Pre-OP SBP (mmHg) | | | | 148.6 ± 20.0 | | | 159.4 ± 18.5 | | < 0.001 | |
| Post-OP SBP (mmHg) | | | | 126.2 ± 13.1 | | | 147.6 ± 17.1 | | < 0.001 | |
| **Δ**SBP (mmHg) | | | | 22.36 ± 20.7 | | | 12.6 ± 22.6 | | 0.002 | |
| Pre-OP DBP (mmHg) | | | | 89.4 ± 14.0 | | | 94.5 ± 12.9 | | 0.007 | |
| Post-OP DBP (mmHg) | | | | 78.5 ± 8.8 | | | 90.1 ± 11.1 | | < 0.001 | |
| **Δ**DBP (mmHg) | | | | 11.0 ± 14.6 | | | 4.6 ± 14.1 | | 0.002 | |
| Pre-OP Serum potassium level (mmol/dl) | | | | 3.44 ± 0.68 | | | 3.67 ± 0.68 | | 0.014 | |
| Log-transformed PAC | | | | 1.70 ± 0.26 | | | 1.70 ± 0.24 | | 0.910 | |
| **Δ**Log-transformed PAC | | | | 0.29 ± 0.34 | | | 0.18 ± 0.34 | | 0.025 | |
| Log-transformed PRA | | | | -0.88 ± 0.75 | | | -0.67 ± 0.75 | | 0.049 | |
| **Δ**Log-transformed PRA | | | | -1.11 ± 0.94 | | | -0.81 ± 1.03 | | 0.035 | |
| Log-transformed ARR | | | | 2.58 ± 0.80 | | | 2.37 ± 0.79 | | 0.059 | |
| **Δ**Log-transformed ARR | | | | 1.41 ± 0.99 | | | 0.98 ± 1.04 | | 0.004 | |
| Pre-OP Number of antihypertensive medication type | | | | 2.0 ± 1.2 | | | 2.6 ± 1.3 | | 0.001 | |
| Post-OP Number of antihypertensive medication type | | | | 0.1 ± 0.5 | | | 1.2 ± 1.1 | | < 0.001 | |
| Change of number of antihypertensive medication type | | | | 1.9 ± 1.2 | | | 1.4 ± 1.5 | | 0.014 | |
| Hypertension history (years) | | | | 6.0 ± 6.2 | | | 9.8 ± 7.4 | | < 0.001 | |
| Pre-OP LVMI (g/m2) | | | | 137.36 ± 41.86 | | | 146.08 ± 39.23 | | 0.124 | |
| Post-OP LVMI (g/m2) | | | | 118.34 ± 32.49 | | | 135.06 ± 34.64 | | < 0.001 | |
| **Δ**LVMI (g/m2) | | | | 19.01 ± 33.13 | | | 11.02 ± 33.32 | | 0.086 | |
| Pre-OP Predicted LVMI (g/m2) | | | | 89.92 ± 17.16 | | | 92.94 ± 15.83 | | 0.190 | |
| Post-OP Predicted LVMI (g/m2) | | | | 77.61 ± 11.54 | | | 89.27 ± 17.65 | | < 0.001 | |
| **Δ**Predicted LVMI (g/m2) | | | | 11.31 ± 14.20 | | | 3.31 ± 17.70 | | 0.001 | |
| Pre-OP Inappropriately excessive LVMI (g/m2) | | | | 47.43 ± 34.56 | | | 53.14 ± 33.03 | | 0.227 | |
| Post-OP Inappropriately excessive LVMI (g/m2) | | | | 39.37 ± 25.70 | | | 45.93 ± 27.69 | | 0.090 | |
| **Δ**Inappropriately excessive LVMI (g/m2) | | | | 9.56 ± 27.06 | | | 6.64 ± 26.69 | | 0.450 | |
| **Supplement Table 2. Clinical characteristics, echocardiographic features, doppler-derived indexes, and change of patients with APA receiving adrenalectomy, with different clinical outcome. (n = 207)** | | | | | | | | | |  |
| Patient characteristics | Clinical cure (n = 95) | | | | Clinical non-cure (n = 112) | | | | |  |
|  | Pre-OP | Post-OP | | p value | Pre-OP | | Post-OP | | p value |  |
| SBP (mmHg) | 148.5 ± 20.2 | 126.2 ± 13.1 | | < 0.001 | 160.1 ± 18.5 | | 147.6 ± 17.1 | | < 0.001 |  |
| DBP (mmHg) | 89.5 ± 14.2 | 78.5 ± 8.8 | | < 0.001 | 94.8 ± 13.1 | | 90.1 ± 11.1 | | 0.001 |  |
| Serum creatinine level (mg/dl) | 0.81 ± 0.32 | 0.94 ± 0.47 | | < 0.001 | 0.99 ± 0.40 | | 1.16 ± 0.77 | | 0.001 |  |
| Serum potassium level (mmol/dl) | 3.49 ± 0.66 | 4.35 ± 0.33 | | < 0.001 | 3.69 ± 0.68 | | 4.29 ± 0.66 | | < 0.001 |  |
| Log-transformed PAC | 1.71 ± 0.26 | 1.42 ± 0.27 | | < 0.001 | 1.70 ± 0.24 | | 1.51 ± 0.26 | | < 0.001 |  |
| Log-transformed PRA | -0.89 ± 0.72 | 0.22 ± 0.51 | | < 0.001 | -0.65 ± 0.73 | | 0.15 ± 0.72 | | < 0.001 |  |
| Log-transformed ARR | 2.60 ± 0.77 | 1.19 ± 0.51 | | < 0.001 | 2.35 ± 0.78 | | 1.37 ± 0.72 | | < 0.001 |  |
| Number of antihypertensive medication type | 2.0 ± 1.3 | 0.1 ± 0.5 | | < 0.001 | 2.6 ± 1.3 | | 1.2 ± 1.1 | | < 0.001 |  |
| Echocardiographic variables |  | | | |  | | | | |  |
| LVEDD (cm) | 4.68 ± 0.39 | 4.60 ± 0.39 | | 0.018 | 4.79 ± 0.51 | | 4.80 ± 0.49 | | 0.873 |  |
| LVESD (cm) | 2.77 ± 0.31 | 2.74 ± 0.33 | | 0.372 | 2.91 ± 0.49 | | 2.90 ± 0.47 | | 0.780 |  |
| IVSD (cm) | 1.15 ± 0.22 | 1.08 ± 0.20 | | < 0.001 | 1.22 ± 0.20 | | 1.16 ± 0.18 | | < 0.001 |  |
| LVPWD (cm) | 1.10 ± 0.19 | 1.01 ± 0.15 | | < 0.001 | 1.15 ± 0.17 | | 1.09 ± 0.14 | | < 0.001 |  |
| RWT | 0.47 ± 0.08 | 0.45 ± 0.09 | | 0.012 | 0.48 ± 0.08 | | 0.46 ± 0.06 | | < 0.001 |  |
| LVEF (%) | 71.27 ± 6.11 | 70.64 ± 6.14 | | 0.416 | 69.21 ± 7.74 | | 69.58 ± 7.70 | | 0.594 |  |
| LVMI (g/m2) | 137.36 ± 41.86 | 118.34 ± 32.49 | | < 0.001 | 146.08 ± 39.23 | | 135.06 ± 34.64 | | 0.001 |  |
| Predicted LVMI (g/m2) | 88.92 ± 16.41 | 77.61 ± 11.54 | | < 0.001 | 92.58 ± 15.96 | | 89.27 ± 17.65 | | 0.058 |  |
| Inappropriately excessive LVMI (g/m2) | 48.93 ± 34.55 | 39.37 ± 25.70 | | 0.001 | 52.57 ± 33.33 | | 45.93 ± 27.69 | | 0.012 |  |

Values are expressed as mean ± SD

ARR, aldosterone–renin ratio; CCB, calcium channel blocker; PAC, plasma aldosterone concentration; PRA, plasma renin activity; IVSD, interventricular septal end diastole thickness; LVEDD, left ventricular end-diastolic diameter; LVEF, left ventricular ejection fraction; LVESD, left ventricular end-systolic diameter; LVMI, left ventricular mass index; LVPWD, left ventricular posterior wall end diastole thickness; RWT, relative wall thickness.
